# Structurally Tunable Graphitized Mesoporous Carbon for Enhancing the Accessibility and Durability of Cathode Pt‐Based Catalysts for Proton Exchange Membrane Fuel Cells

**DOI:** 10.1002/smsc.202400016

**Published:** 2024-05-19

**Authors:** Mingjuan Wu, Zihan Meng, Yifei Xiong, Haining Zhang, Aojie Zhang, Hao Zhang, Liyan Zhu, Haibo Tang, Tian Tian, Haolin Tang

**Affiliations:** ^1^ State Key Laboratory of Advanced Technology for Materials Synthesis and Processing Wuhan University of Technology Wuhan 430070 China; ^2^ National Energy Key Laboratory for New Hydrogen‐Ammonia Energy Technologies Foshan Xianhu Laboratory Foshan 528200 China

**Keywords:** catalyst durabilities, graphitized mesoporous carbons, oxygen reduction reactions, proton exchange membrane fuel cells, reactant accessibilities

## Abstract

Low Pt utilization and intense carbon corrosion of cathode catalysts is a crucial issue for high‐efficiency proton exchange membrane fuel cells due to the highly demanded long‐term durability and less acquisition/application cost. Herein, structurally tunable graphitized mesoporous carbon (GMC) is obtained by direct high‐temperature pyrolysis and in situ‐controlled mesopore formation; the structure‐optimized GMC1300‐1800 exhibits a mesopore size of 7.54 nm and enhanced corrosion resistance. Functionalized GMC1300‐1800 is loaded with small‐sized Pt nanoparticles (NPs) (1.5 nm) uniformly by impregnation method to obtain Pt/GMC1300‐1800 and form an “internal platinum structure” to avoid sulfonic acid groups poisoning as well as ensure O_2_/proton accessibility. Hence, the electrochemically active surface area (ECSA) of Pt/GMC1300‐1800 reaches 106.1 m^2^ g^−1^_Pt_, while mass activity and specific activity at 0.9 V are 2.1 and 1.4 times those of commercial Pt/C, respectively. Notably, the ECSA decay is less than 17% for both 30 000 cycles’ accelerated durability tests (ADTs) of Pt attenuation and carbon attenuation. Accordingly, the optimized mesoporous structure of GMC1300‐1800 significantly decreases the coverage of sulfonic acid groups on Pt NPs, leading to the highest peak power density in the single‐cell test. Density functional theory calculations demonstrate the synergistic effect between graphitization and mesoporosity on enhancing the accessibility and durability of the catalysts.

## Graphical abstract

Structurally tunable graphitized mesoporous carbon supports are synthesized by a direct pyrolysis method and in situ‐controlled mesopore formation. The distinction in mesopore size of carbon support affects the size, dispersion, and location of Pt nanoparticles. Meanwhile, the synergistic effect between graphitization and mesoporous structure of carbon support enhances the reactant accessibility, antipoisoning, and durability of the oxygen reduction reaction catalysts.

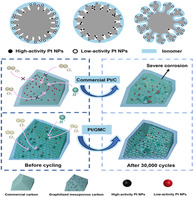

## 1 Introduction

In response to the global demand for carbon neutrality, proton exchange membrane fuel cells (PEMFCs) have attracted considerable attention as one of the promising candidates for the next generation of power sources with environmental friendliness and high energy density.^[^ ^1^ ^]^ Although numerous impressive advances have been employed in recent years, there are still significant obstacles that need to be overcome for large‐scale applications, such as the high cost of acquisition and utilization as well as insufficient long‐term durability.^[^ ^2^ ^]^ The catalyst is an essential element of PEMFCs, acting as the catalytic active core of membrane electrode assembly (MEA).^[^ ^3^ ^]^ At present, the performance of catalysts has been greatly influenced by the dispersion and existence form of noble metal nanoparticles (NPs) on carbon support as well as the structure evolution of noble metal NPs and carbon support.^[^ ^4^ ^]^ Therefore, one of the crucial solutions to these issues is to rationally design a robust catalyst support for noble metal NPs loading with high utilization.

Currently, the loading of Pt NPs on carbon support is still the mainstream cathode catalyst for PEMFC.^[^ ^5^ ^]^ The poor performance of Pt/C catalysts is attributed as follows: 1) The sulfonic acid groups with high proton conductivity in ionomers tend to adsorb on Pt NPs, resulting in low utilization of Pt and inferior accessibility to O_2_;^[^ ^6^ ^]^ 2) Low dispersion in light of limited support surface leads to large‐sized Pt NPs with low Pt utilization and diversity in surface energy, resulting in the Pt NPs agglomeration and poor durability;^[^ ^7^ ^]^ and 3) To enhance the efficiency of PEMFCs, the corrosion degree of carbon support increases significantly with the operating voltage rises, unfavorable carbon corrosion gives rise to agglomeration/detachment of Pt NPs and limited durability.^[^ ^8^ ^]^ Therefore, the nanostructure of carbon support is essential for highly dispersed Pt NPs without ionomer poison but ensuring the reactant accessibility to reduce the cost of PEMFC acquisition, while the property of carbon support is crucial for stable operation with high‐efficiency conditions to reduce the cost of PEMFC application and endow attractive useful lifetime.

Numerous efforts have been devoted to improving the performance of cathode Pt‐based catalysts by modifying the carbon support structure, but it is difficult to achieve comprehensive performance innovation.^[^ ^9^ ^]^ For example, an appropriate pore structure can increase the exposure of active sites to promote the utilization of Pt, thus decreasing the platinum group metal loading.^[^ ^10^ ^]^ In this sense, porous carbon support with large specific surface area is usually chosen as the catalyst support for Pt NP loading, such as carbon nanotubes,^[^ ^11^ ^]^ graphene,^[^ ^12^ ^]^ carbon nanofibers,^[^ ^13^ ^]^ and modified carbon black.^[^ ^14^ ^]^ However, high accessibility of O_2_ and proton is difficult to be fully realized by the porous structure alone; thus, pore size and transport channels of porous carbon are under the research spotlight. Yarlagadda et al.^[^ ^15^ ^]^ proposed that the ideal support was preferably mesoporosity to accommodate Pt NPs and protect them from the direct adsorption of ionomers but still allows proton and O_2_ to approach them reasonably. Moreover, the depth of mesopores and the curvature should not be too large, this kind of carbon support is called accessible mesoporous carbon (MC). Xie et al.^[^ ^16^ ^]^ constructed a spongy porous carbon with 3–4 nm mesopores and demonstrated that the “internal Pt structure” for cathode catalyst for PEMFCs could boost catalytic activity and avoid the toxic effect of ionomers on Pt NPs. Although the confinement effect of mesopores reduced the agglomeration and dissolution of Pt NPs, it is suggested that the increased area exposed to high O_2_ concentration and humidity led to intensified corrosion of carbon support; thus, the durability of the catalyst would be the next direction in their work.

It is well known that graphitized structure can improve the oxidation corrosion resistance of carbon support to enhance the electrochemical durability of catalysts.^[^ ^17^ ^]^ However, specific surface area and pore size of support invariably decrease after graphitization, coupled with sluggishness, and hydrophobic surfaces are unfavorable for aqueous solution impregnating, thus making it difficult to uniformly disperse small‐sized Pt NPs and impede proton accessibility of Pt NPs inside the mesopores.^[^ ^18^ ^]^ Qiao et al. improved the dispersion of Pt NPs and increased the durability of the catalyst by 3D porous graphitized carbon support, which was a combination of Mn species and pyrolysis of 3D polymer hydrogels.^[^ ^19^ ^]^ The balance between graphitization and hierarchical porosity makes it favorable for the comprehensive performance of catalysts, but it should be noted that the method of carbon support preparation was complicated with low reproducibility. The relationships between mesopore size, dispersion of Pt NPs, reactant accessibility, and catalytic activity/durability had not been thoroughly discussed. Therefore, the development of the structurally tunable catalyst support to balance three major factors (uniformity of loading small‐sized Pt NPs, high accessibility of O_2_/proton, and anticorrosion ability of carbon support) is a top priority to enhance the comprehensive performance (activity, accessibility, and durability) of PEMFC cathode catalyst. Besides, the simplicity and optimization of the preparation process are necessary prerequisites for large‐scale application in the future.

In this work, structurally tunable graphitized mesoporous carbons (GMC) were synthesized through a direct pyrolysis method with controllable in situ mesopore formation that can be easily amplified manufacture, and the effects of mesopore sizes on the Pt NPs loading as well as on the activity and accessibility of catalysts were systematically explored. Three Pt‐based catalysts Pt/GMC*x*‐1800 (*x* = 1100, 1300, and 1500) were obtained by impregnation methods, transmission electron microscopy (TEM) images and Brunauer–Emmett–Teller (BET) results revealed that mesopores of GMC1300‐1800 were most suitable for aqueous solution impregnation to uniformly load small‐sized Pt NPs and make it favorable for O_2_ and proton rapidly arriving at internal Pt NPs without poisoning by ionomer, and electrochemical activity far exceeded that of Pt/C‐JM. Graphitized structure improved the durability of the as‐prepared catalyst with an ECSA decay rate of less than 17% after 30 000 cycles of Pt attenuation or 30 000 cycles of carbon attenuation tests. Meanwhile, it was confirmed in MEA that the “internal Pt structure” of GMC1300‐1800 decreased the coverage of sulfonic acid groups on Pt NPs. The comprehensive performance exhibited by the as‐prepared catalysts over Pt/C‐JM was also explained by the more stable adsorption configuration of Pt clusters and O_2_ on GMC in density functional theory (DFT) calculations.

## 2 Results and Discussion

### 2.1 Structural Characterization

**Figure 1:**
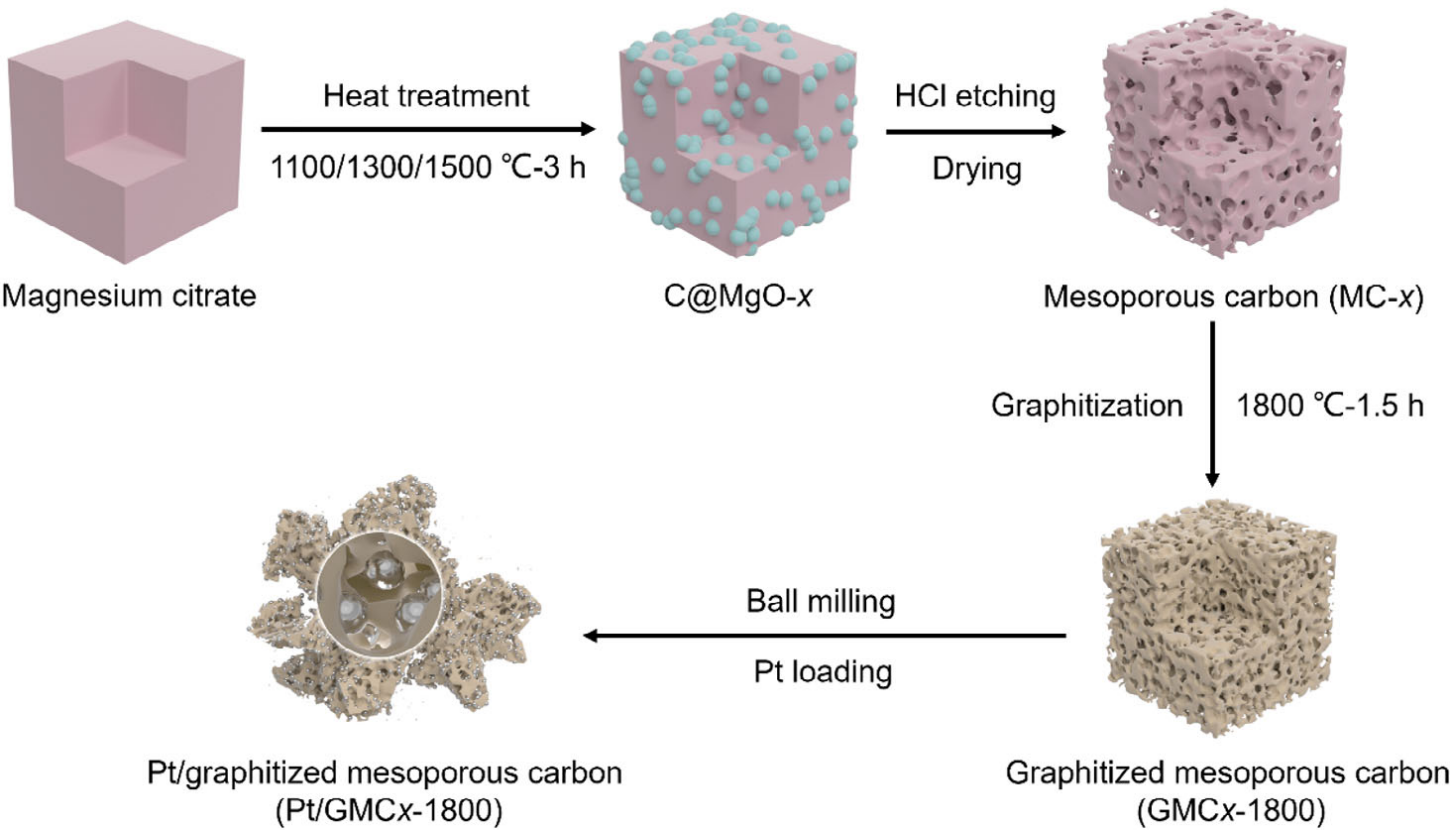
Schematic preparation of magnesium citrate‐derived GMC and Pt/GMC catalysts.

The preparation schematic diagram of as‐synthesized catalysts is shown in **Figure** **1**. First, magnesium citrate hydrate was in situ transformed into MgO templates with tunable sizes encapsulated by carbon (C@MgO) through a direct pyrolysis at different temperatures under argon atmosphere. After HCl etching, the in situ‐generated MgO templates were removed and MC with controllable pore sizes was obtained. GMC was formed after graphitization, and its particle size and surface chemical state were also altered by ball milling and functionalization. Then, the catalysts with a Pt concentration of 20 wt% were obtained by wet impregnation method.

Scanning electron microscopy (SEM) images of C@MgO‐*x* (*x* = 1100, 1300, and 1500) with different sizes of in situ‐formed MgO NPs are shown in Figure S1, Supporting Information. SEM observations indicated that the size of MgO NPs (circled in orange, Figure S1a–c, Supporting Information) grew with the increasing temperature. The X‐Ray diffraction (XRD) spectra of C@MgO‐*x* are shown in Figure S1d, Supporting Information, with diffraction peaks corresponding to MgO at 2*θ* ≈42.9° and 62.3°. The resulting average sizes of MgO crystallites obtained by the Scherrer equation were 5.6, 8.2, and 11.7 nm,^[^ ^20^ ^]^ respectively, which were in agreement with SEM results. TEM images of MC‐*x* formed after HCl etching are shown in Figure S2a–c, Supporting Information. It can be clearly observed that the size of the mesopores corresponded to that of the MgO templates (circled in yellow). The XRD spectrum of MC‐*x* (Figure S2d, Supporting Information) confirmed the disappearance of the characteristic peaks of MgO, indicating that the templates had been completely removed during the acid etching process. In addition, comparing the Raman spectra of MC‐*x* with GMC*x*‐1800 (Figure S3, Supporting Information), it can be seen that GMC*x*‐1800 showed a 2D band peak at 2680 cm^−1^ caused by the strong vibration of neighboring carbon atoms in the opposite direction in the graphitized carbon network plane, implying the generation of graphitized structures.^[^ ^21^ ^]^ Particle sizes of GMC*x*‐1800 (Figure S4a–c, Supporting Information) were in the micrometer‐size range before ball milling, while the agglomeration degree and particle size became greater with increasing heat treatment temperature (d_GMC1100‐1800_ < d_GMC1300‐1800_ < d_GMC1500‐1800_). After ball milling for 36 h, the particle size of three kinds of graphitized carbons was reduced below 500 nm. Figure S4d–i (Supporting Information) shows the corresponding SEM images, which confirmed the validity of ball milling to regulate the diameter of carbon support from micrometer grade to nanograde.

**Figure 2:**
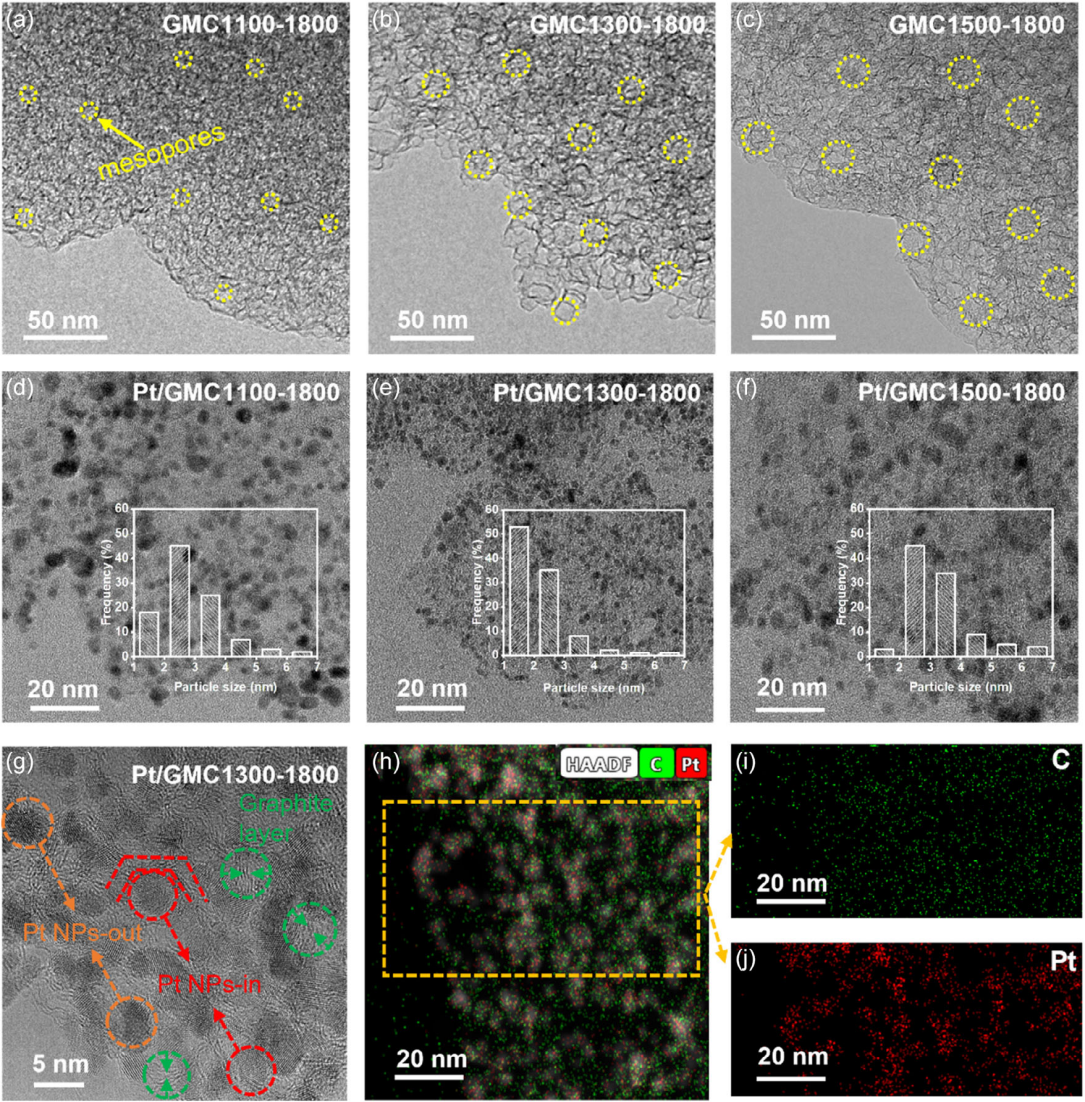
TEM images of a) GMC1100‐1800, b) GMC1300‐1800, c) GMC1500‐1800, d) Pt/GMC1100‐1800, e) Pt/GMC1300‐1800, and f) Pt/GMC1500‐1800. g) HRTEM images, h) HAADF‐STEM images, i) C elemental mapping images, and j) Pt elemental mapping images of Pt/GMC1300‐1800.

TEM images of GMC*x*‐1800 exhibited that the pore diameter had shrunk to a certain extent compared with MC‐*x* (**Figure** **2**a–c), which may be attributed to the slight collapse of the pore structure during graphitization.^[^ ^22^ ^]^ The collapsed degree and the reduction proportion of pore size increased with the temperature rise. Three Pt‐based catalysts, Pt/GMC*x*‐1800 (*x* = 1100, 1300, and 1500), were prepared based on GMC*x*‐1800 through the platinum nitrate solution impregnation method, and the respective TEM images are shown in Figure 2d–f. After careful observation, it was found that Pt NPs in Pt/GMC1300‐1800 were the most uniformly distributed and the smallest, whereas a certain degree of agglomeration was observed in Pt/GMC1100‐1800 and Pt/GMC1500‐1800. This was probably due to the fact that the mesopore size of GMC1100‐1800 was too small to allow abundant platinum nitrate solution to enter during the impregnation process, hence mainly clustered on the surface of carbon support. In contrast, the mesopore size of GMC1500‐1800 was wide enough for aqueous solution impregnating, so the reduced Pt NPs mostly aggregated inside the mesopores. However, the medium mesopore size of GMC1300‐1800 led to a proper amount of platinum nitrate solution entering and reducing inside mesopores with sufficient proton accessibility as well as other precious metal precursors uniformly dispersed in the surface with less agglomeration. To further confirm the hypothesis, high resolution transmission electron microscope (HRTEM) images of Pt/GMC1300‐1800 were performed and analyzed (Figure 2g), where a clear graphite layer was circled in green. The hollow circular graphite layer structure was regarded as a mesopore, and platinum NPs located in this circular region could form an “inner Pt structure.”^[^ ^23^ ^]^ Pt NPs in the interior region of the mesopore were named Pt NPs‐in (circled in red), and those located in the exterior region were called Pt NPs‐out (circled in orange). It was obvious that mesopore size influenced the distribution of Pt NPs, but the difference between Pt NPs inside and outside the mesopores in three as‐prepared catalysts could not be quantified by TEM images alone, so the coverage of sulfonate groups tested in single cell was further discussed. High‐angle annular dark‐field (HAADF)–scanning transmission electron microscopy (STEM) images of Pt/GMC1300‐1800 as provided in Figure 2h–j confirmed that the Pt element was uniformly dispersed throughout the catalyst.

**Figure 3:**
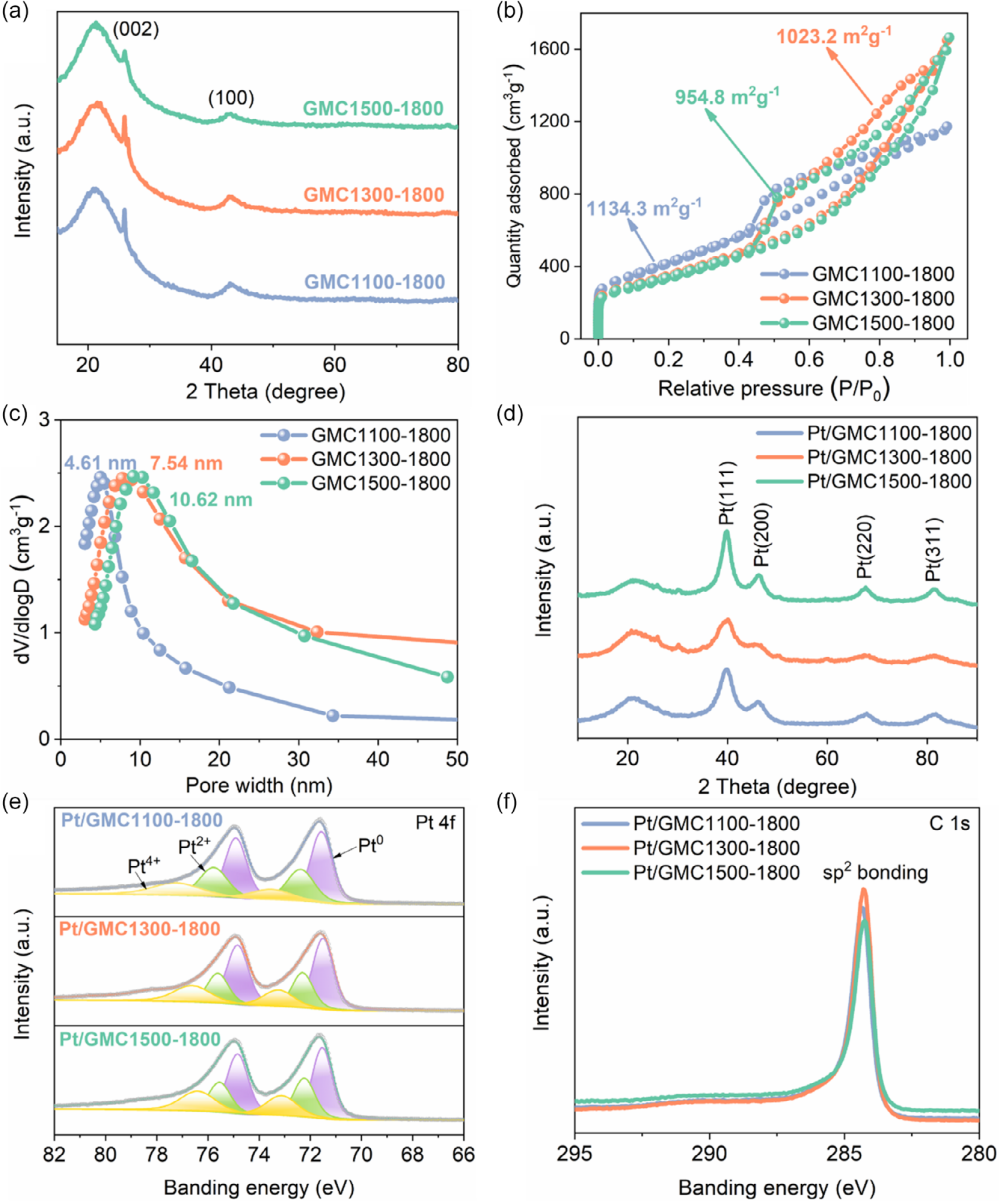
a) XRD patterns, b) N_2_ adsorption/desorption isotherms, and c) pore‐size distribution of GMC*x*‐1800. d) XRD patterns, e) Pt 4f high‐resolution XPS spectra, and f) C 1*s* high‐resolution XPS spectra of Pt/GMC*x*‐1800.

**Figure** **3**a shows the XRD spectra of GMC*x*‐1800, the diffraction peaks at 26° and 43° corresponded to (002) and (100) crystal planes of graphitized carbon, which did not present in the aforementioned XRD spectra of MC‐*x*, further indicating the transformation from disordered structures to robust graphitized structures.^[^ ^5^ ^]^ The average pore size and specific surface area of GMC*x*‐1800 were also evaluated from N_2_ adsorption–desorption isotherms. All the samples showed typical type‐IV adsorption–desorption curves with H3‐type isotherms hysteresis loops (Figure 3b), which indicated that the as‐prepared carbon support featured mesoporous structure after graphitization,^[^ ^24^ ^]^ in good agreement with the TEM images. GMC1100‐1800, GMC1300‐1800, and GMC1500‐1800 showed specific surface area of 1134.3, 1023.2, and 954.8 m^2^ g^−1^ as well as the concentrated pore sizes of 4.61, 7.54, and 10.62 nm, respectively (Figure 3c, Table S1, Supporting Information). It is suggested that carbon support with high specific surface area can inhibit the aggregation of Pt NPs and provide more surface area of available Pt NPs loading.^[^ ^25^ ^]^ Meanwhile, numerous researches have demonstrated that the ionomer cannot access small‐sized mesopores, so the mesoporosity of carbon support is also essential for protecting Pt NPs from sulfonic acid groups poisoning to achieve favorable electrocatalytic activity with satisfactory O_2_ and proton accessibility.^[^ ^26^ ^]^

The crystal structure of as‐synthesized catalysts was analyzed by XRD given in Figure 3d, where the signals at 39.8°, 46.1°, 67.8°, and 81.4° can be indexed to the Pt (111), (200), (220), and (311) facets, respectively.^[^ ^27^ ^]^ The results suggested that all samples showed four typical characteristic peaks of the face‐centered‐cubic structure of Pt, indicating a high degree of crystallinity. Meanwhile, the average diameters of Pt crystallites on Pt/GMC1100‐1800, Pt/GMC1300‐1800, and Pt/GMC1500‐1800 were calculated as 2.3, 1.5, and 2.9 nm according to the Scherrer equation (Table S2, Supporting Information), which was in agreement with the TEM images. The Pt contents collected from inductively coupled plasma (ICP)‐optical emission spectrometry (OES) were 21.8, 21.1, and 22.3 wt% for Pt/GMC1100‐1800, Pt/GMC1300‐1800, and Pt/GMC1500‐1800, respectively (Table S2, Supporting Information).

X‐Ray photoelectron spectroscopy (XPS) spectroscopy was used to analyze the near‐surface elemental composition and valence states of as‐prepared catalysts. The Pt 4*f* signal of Pt/GMC*x*‐1800 was deconvoluted into three contributions (Figure 3e), which can be assigned to the Pt^0^, Pt^2+^, and Pt^4+^ states. The state with low binding energies (71.5 and 74.9 eV) corresponded to Pt^0^,^[^ ^28^ ^]^ and the percentages of Pt^0^ in Pt/GMC1100‐1800, Pt/GMC1300‐1800, and Pt/GMC1500‐1800 were 51.56%, 54.13%, and 49.39%, respectively. Generally, Pt^0^ was the predominant species on the surface of catalysts and it was favorable for catalytic activity. It was found that Pt/GMC1300‐1800 contained the highest proportion of Pt^0^, indicating that an appropriate graphitized mesoporous structure could facilitate the catalytic activity. The C 1*s* spectrogram is shown in Figure 3f, where *sp* ^2^ binding peak appeared near the binding energy of 284.28 eV, confirming the transformation of carbon support from a disordered state to a graphitic ordered structure.^[^ ^29^ ^]^ This provides the possibility of maintaining robust durability for carbon supports in water–oxygen environments with high operating voltages for high‐efficiency PEMFC.

### 2.2 Electrochemical Catalytic Performance

**Figure 4:**
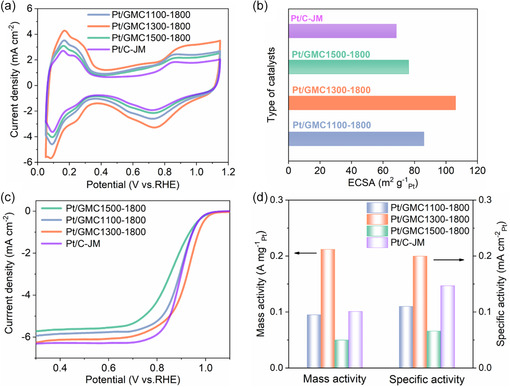
Initial electrocatalytic performance of Pt/GMC*x*‐1800 and Pt/C‐JM for ORR. a) CV comparison in the potential range of 0.05–1.15 V at 50 mV s^−1^. b) ECSA values calculated from the hydrogen adsorption peak area of corresponding CV curves. c) LSV comparison in the potential range of 0.05–1.15 V at 10 mV s^−1^. d) MA and SA values at 0.9 V after IR correction.

The critical properties of catalysts were compared to get a better understanding of the electrochemical performance, and the influence of graphitized mesoporous structure on the catalytic performance was analyzed. As presented in **Figure** **4**a, the hydrogen adsorption peak area of the corresponding curve for Pt/GMC1300‐1800 was the largest with the calculated ECSA value of 106.1 m^2^ g^−1^. The ECSAs of Pt/GMC1100‐1800, Pt/GMC1500‐1800, and Pt/C‐JM were calculated as 86.0, 76.3, and 68.5 m^2^ g^−1^, respectively (Figure 4b). Notably, the ECSA of Pt/GMC*x*‐1800 presented higher than that of Pt/C‐JM, suggesting that as‐prepared catalysts possessed a larger area of accessible active sites relying on the mesopore structure.^[^ ^30^ ^]^ Moreover, Pt/GMC1500‐1800 had the smallest ECSA among the three as‐synthesized catalysts, which may relate to the reduced specific surface area and large‐sized Pt NPs. Figure 4c shows the linear scanning voltammetry (LSV) curves of as‐prepared catalysts and commercial Pt/C‐JM. Pt/GMC1300‐1800 exhibits a notably higher onset potential than other catalysts, mainly assigned to the suitable pore size for internal Pt loading with a low degree of ionomer poisoning and high reactant accessibility.^[^ ^31^ ^]^ Pt/GMC1300‐1800 also showed the half‐wave potentials of 0.918 V, much higher than that of Pt/C‐JM (0.899 V). The mass activity (MA) and specific activity (SA) values of all catalysts are displayed in Figure 4d. Due to the proper pore size of GMC1300‐1800 to afford aqueous solution access, a mass of small‐sized Pt NPs uniformly located inside the mesopore without ionomer poisoning, the relatively large mesopores also facilitate O_2_ transfer and ensure proton conduct through impregnated water inside the mesopores, leading to the superior catalytic activity of Pt/GMC1300‐1800 (Table S3, Supporting Information). To underline the significance of the findings, we compared the catalyst with the state‐of‐the‐art Pt‐based oxygen reduction reaction (ORR) catalysts (Table S4, Supporting Information).^[^ ^32^ ^]^

**Figure 5:**
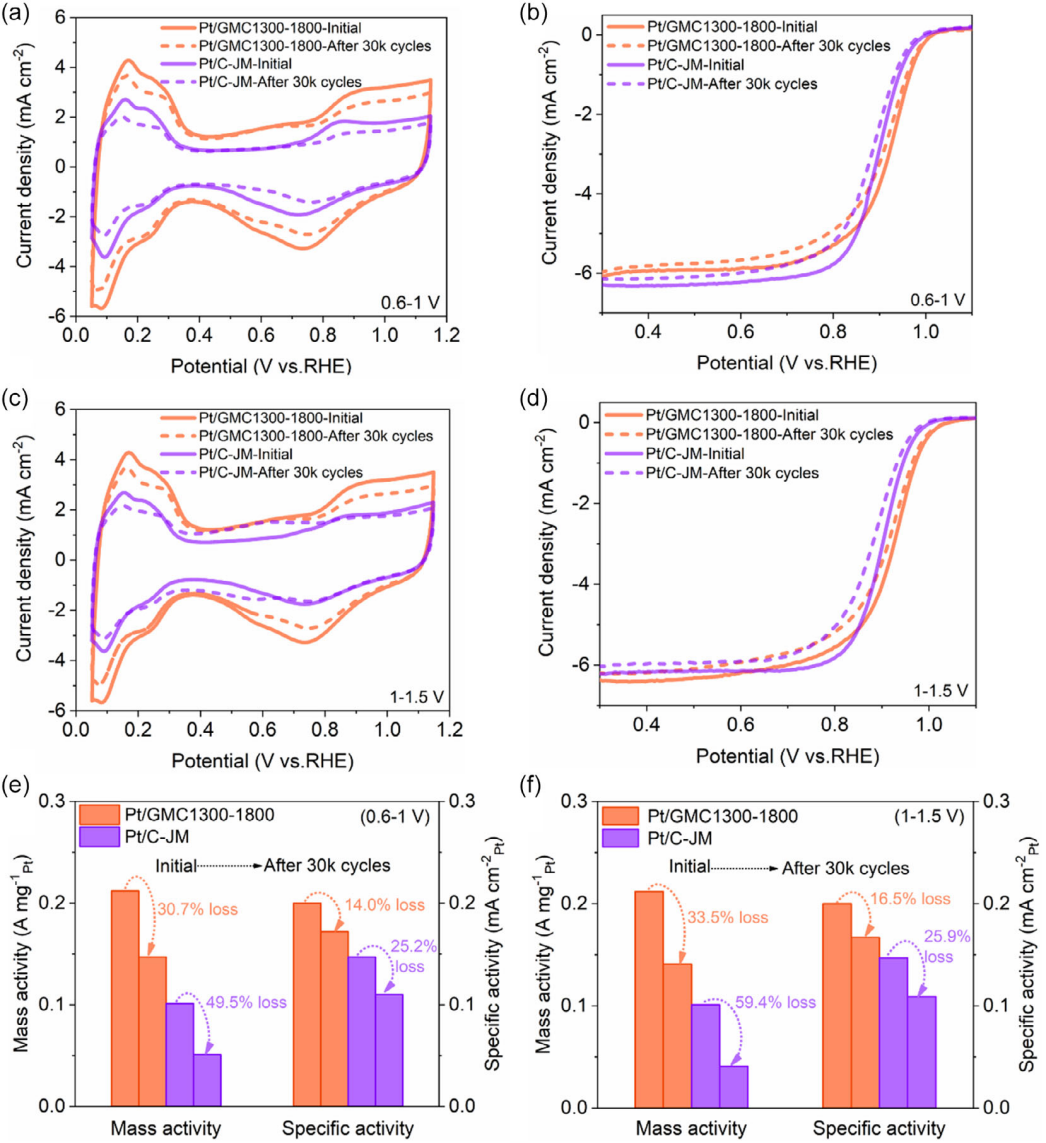
Pt attenuation test (0.6–1 V) of Pt/GMC1300‐1800 and Pt/C‐JM: a) CV curves at 50 mV s^−1^ and b) LSV curves at 10 mV s^−1^ before and after 30 000 cycles. Carbon support attenuation test (1–1.5 V) of Pt/GMC1300‐1800 and Pt/C‐JM: c) CV curves at 50 mV s^−1^ and d) LSV curves at 10 mV s^−1^ before and after 30 000 cycles. Comparison of the loss of MA and SA during e) Pt attenuation test and f) carbon support attenuation test.

**Figure** **5**a illustrates the cyclic voltammetry (CV) curves before and after the ADT performed at the voltage range of 0.6–1 V, and it can be concluded that the retention of ECSA for Pt/GMC1300‐1800 and Pt/C‐JM during Pt attenuation was 80.7% and 67.6%, respectively (Tables S5 and S6, Supporting Information). Concurrently, the LSV curves presented in Figure 5b were all shifted to a larger overpotential after 30 K cycles, and the half‐wave potential was negative shifted by 5 and 14 mV for Pt/GMC1300‐1800 and Pt/C‐JM, suggesting that Pt/GMC1300‐1800 exhibited better ORR stability. The ADTs for carbon support were performed in a high potential range of 1–1.5 V (Figure 5c,d), where the retention of ECSA after 30 K cycles was 79.6% for Pt/GMC1300‐1800 and 54.5% for Pt/C‐JM, with corresponding half‐wave potentials decreased by 8 and 16 mV, respectively.

After normalization for the Pt attenuation test, the MA decay rates of Pt/GMC1300‐1800 and Pt/C‐JM were 30.7% and 49.5%, while the SA decay rates were 14.0% and 25.2%, respectively (Figure 5e). Meanwhile, after 30 K cycles of carbon support attenuation tests, the MA decay rates of Pt/GMC1300‐1800 and Pt/C‐JM were 33.5% and 59.4%, while the MA decay rates were 16.5% and 25.9%, respectively (Figure 5f). The attenuation process for every 10 K cycles is shown in Figure S5 (Pt attenuation, Supporting Information) and Figure S6 (carbon support attenuation, Supporting Information). Thanks to the combination of highly graphitized carbon support and its mesoporous nanostructure, Pt/GMC1300‐1800 displayed more robust durability than that of Pt/C‐JM, especially in high‐voltage working conditions. On the one hand, it is well known that the poor durability of catalysts is due to the coarsening of Pt NPs, mainly caused by the oxidative corrosion of carbon support during working conditions such as high‐voltage operation or start–stop cycling.^[^ ^33^ ^]^ The confinement effect of mesopores reduces the detachment and agglomeration of internal Pt NPs and ensures a relatively high ECSA retention, thus improving the stability of the catalyst.^[^ ^34^ ^]^ On the other hand, graphite lattice from graphitized carbon support effectively inhibits oxidation and ensures interaction between Pt NPs and carbon support, impeding detachment and agglomeration of Pt NPs.^[^ ^35^ ^]^

**Figure 6:**
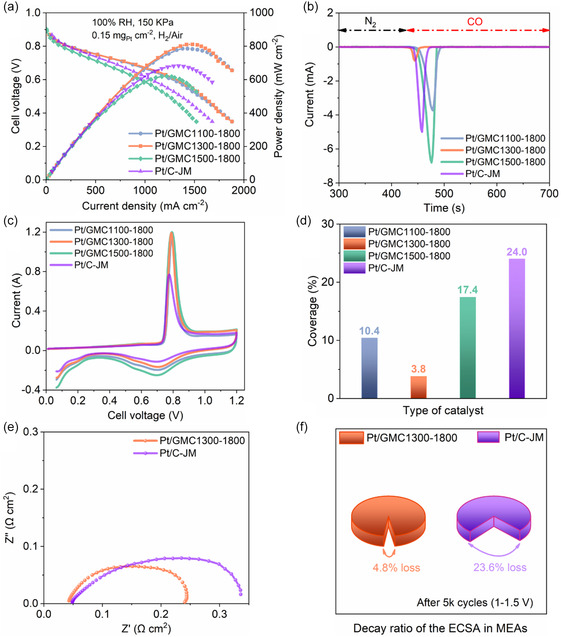
a) Polarization curves and power densities. b) Current–time curves of CO displacement. c) CV curves during the CO stripping process and d) sulfonic acid group coverage on Pt NPs of MEAs with Pt/GMC*x*‐1800 and Pt/C‐JM. e) Impedance spectra and f) decay rate of ECSA in MEAs with Pt/GMC1300‐1800 and Pt/C‐JM.

Given the significant differences in conditions between conventional rotating disc electrode (RDE) and actual PEMFCs, the as‐prepared catalysts were meticulously evaluated in MEA with a practical H_2_/air environment and compared to commercial Pt/C.^[^ ^36^ ^]^ **Figure** **6**a shows the polarization curves and power densities of MEA; the relatively low open‐circuit voltage is mainly attributed to the relatively high hydrogen crossover values of homemade PEM, which hardly affect activation/Ohmic/concentration polarization for comparison.^[^ ^37^ ^]^ Possessing a portion of high‐activity Pt NPs with satisfactory O_2_ and proton accessibility, Pt/GMC1300‐1800 showed a voltage of 0.67 V at the current density of 1,000 mA cm^−2^, much higher than that of Pt/C‐JM (0.62 V@1,000 mA cm^−2^). It is suggested that Pt/GMC1300‐1800 displayed the highest peak power density of 823 mW cm^−2^, which was highly consistent with the result in the RDE test.

CO displacement chronoamperometry was employed to further evaluate the local coverage of sulfonic acid groups on Pt NPs.^[^ ^38^ ^]^ Figure 6b shows the current–time diagram of CO displacement, and the different amounts of electricity on the Pt surface can be observed, indicating that the total amount of sulfonic acid groups adsorbed on the Pt surface varied depending on the nanostructure of carbon support.^[^ ^39^ ^]^ The CO oxidative stripping CV curves are shown in Figure 6c and the coverage of the sulfonic acid groups on the surface of Pt is presented in Figure 6d. It should be noted that the coverage of sulfonic acid groups on Pt/GMC1300‐1800 is the lowest, further demonstrating a relatively high percentage of active Pt NPs.^[^ ^16^ ^]^ The coverages of sulfonic acid groups on Pt/GMC1100‐1800, Pt/GMC1500‐1800, and Pt/C‐JM were 10.4, 17.4, and 24.0%, respectively, which was in high agreement with the analysis of TEM images regarding the dispersion and location of Pt NPs in as‐prepared catalysts. The mesopore size of GMC1100‐1800 was too small for Pt precursor solution and ionomers to enter the interior, so a large number of sulfonic acid groups were adsorbed on Pt NPs located on the outer surface of carbon support. GMC1300‐1800 had moderate mesopores, which allowed aqueous solution inwardly and avoided ionomers entering to protect inside Pt NPs from sulfonic acid groups adsorption with high O_2_ and proton accessibility.^[^ ^40^ ^]^ The mesopore size of GMC1500‐1800 was too wide, allowing a large number of Pt precursor reduction and ionomers access; adsorption of sulfonic acid groups took place both on the outer surface and inside the mesopores. Based on the micropore‐dominated carbon support of Pt/C‐JM, Pt NPs were dispersed on the surface and the sulfonic acid group was directly adsorbed on the particles, resulting in the highest coverage.

Figure 6e illustrates the impedance spectrum of MEA and the semicircle diameter of Pt/GMC1300‐1800 was lower than that of Pt/C‐JM. This may be not only related to the increase in electrical conductivity and more robust structure of carbon support after graphitization,^[^ ^41^ ^]^ but also to the superior O_2_ and proton transport channels due to the optimized mesoporosity.^[^ ^42^ ^]^ In general, the impedance of all O_2_ transport media in PEMFC is interdependent, so it reflected that Pt/GMC1300‐1800 had a better reactant transport capacity. Figure 6f shows the decay rate of ECSA in MEAs after 5 K cycles at the potential range of 1–1.5 V. It was clearly found that the ECSA attenuation rate of MEA with Pt/C‐JM was around 5 times that of MEA with Pt/GMC1300‐1800, and the ECSA attenuation rate of MEA with Pt/GMC1100‐1800 (Figure S7, Supporting Information) was also higher than that of MEA with Pt/GMC1300‐1800, indicating the robust durability of Pt/GMC1300‐1800.^[^ ^43^ ^]^ To gain further insight into the relationship between catalyst durability and structure evolution, the morphology of the catalysts before and after attenuation tests was characterized by TEM (Figure S8, Supporting Information).^[^ ^44^ ^]^ The corresponding TEM images exhibited that the Pt NPs size in Pt/GMC1300‐1800 slightly increased while heavily coarsened and agglomerated in Pt/C‐JM after the ADT. This further demonstrated that graphitized mesoporous structure could not only increase the stability of carbon support, but also hinder the agglomeration of Pt NPs.

### 2.3 DFT Calculations

It is commonly known that the first step of an electrochemical reaction is the contact of reactants with the catalyst surface,^[^ ^45^ ^]^ and the optimal adsorption configuration is precisely a prerequisite for the subsequent reaction,^[^ ^46^ ^]^ so it is crucial to take the adsorption configuration into thorough consideration. The theoretical background of this work is that the adsorption of sulfonic acid groups on Pt NPs leads to low activity and also makes it difficult for O_2_ to reach the active sites through ionomer, reducing the effective utilization of the catalyst.^[^ ^47^ ^]^ Simultaneously, oxidative corrosion of carbon support alters the bonding of Pt NPs and leads to Pt NP agglomeration/detachment as well as ultimately poor device durability.^[^ ^48^ ^]^ Therefore, we developed a series of GMC to overcome these challenges; it is crucial to employ theoretical simulations to realize the adsorption process of O_2_ and Pt clusters on the surface of carbon support with different structures.

**Figure 7:**
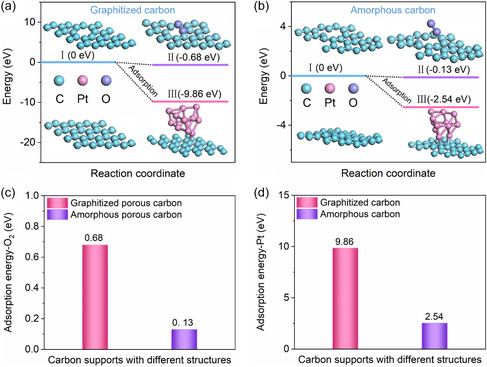
Adsorption variation processes of O_2_ molecules and Pt clusters on a) graphitized carbon and b) amorphous carbon. Comparison of adsorption energies of c) O_2_ molecules and d) Pt clusters on carbon support with different structures.

The transition state of Pt clusters and O_2_ molecules adsorbed on graphitized carbon is shown in **Figure** **7**a, while the theoretical model of amorphous carbon consisting of multiple disordered chains in Figure 7b was formed based on molecular dynamics simulations.^[^ ^49^ ^]^ Figure 7c shows that the adsorption energies of O_2_ molecules on graphitized porous carbon and amorphous porous carbon (E_ad1_‐O_2_, E_ad2_‐O_2_) were 0.68 and 0.13 eV, while O_2_ molecules were more likely to adsorb near the nanopore in the model of the graphitized carbon. The adsorption energies of Pt clusters on graphitized carbon and amorphous carbon (E_ad1_‐Pt, E_ad2_‐Pt) were 9.86 and 2.54 eV, respectively (Figure 7d). Consequently, the adsorption of O_2_ molecules and Pt clusters on graphitized carbon was stronger than that on amorphous carbon, which was instructive for practical experiments. First, the graphitized structure resulted in a more affinity of Pt clusters,^[^ ^50^ ^]^ which was reflected in the robust durability of the catalyst. In addition, the porous graphitized structure had a similar “defect‐inducing” effect on the adsorption configurations, which increased the targeted adsorption of O_2_ molecules inside the pores and reflected in the accessibility and activity of the catalysts.^[^ ^51^ ^]^ In general, DFT calculations confirmed that the balance between graphitization and porosity could boost the comprehensive performance of catalysts.

**Figure 8:**
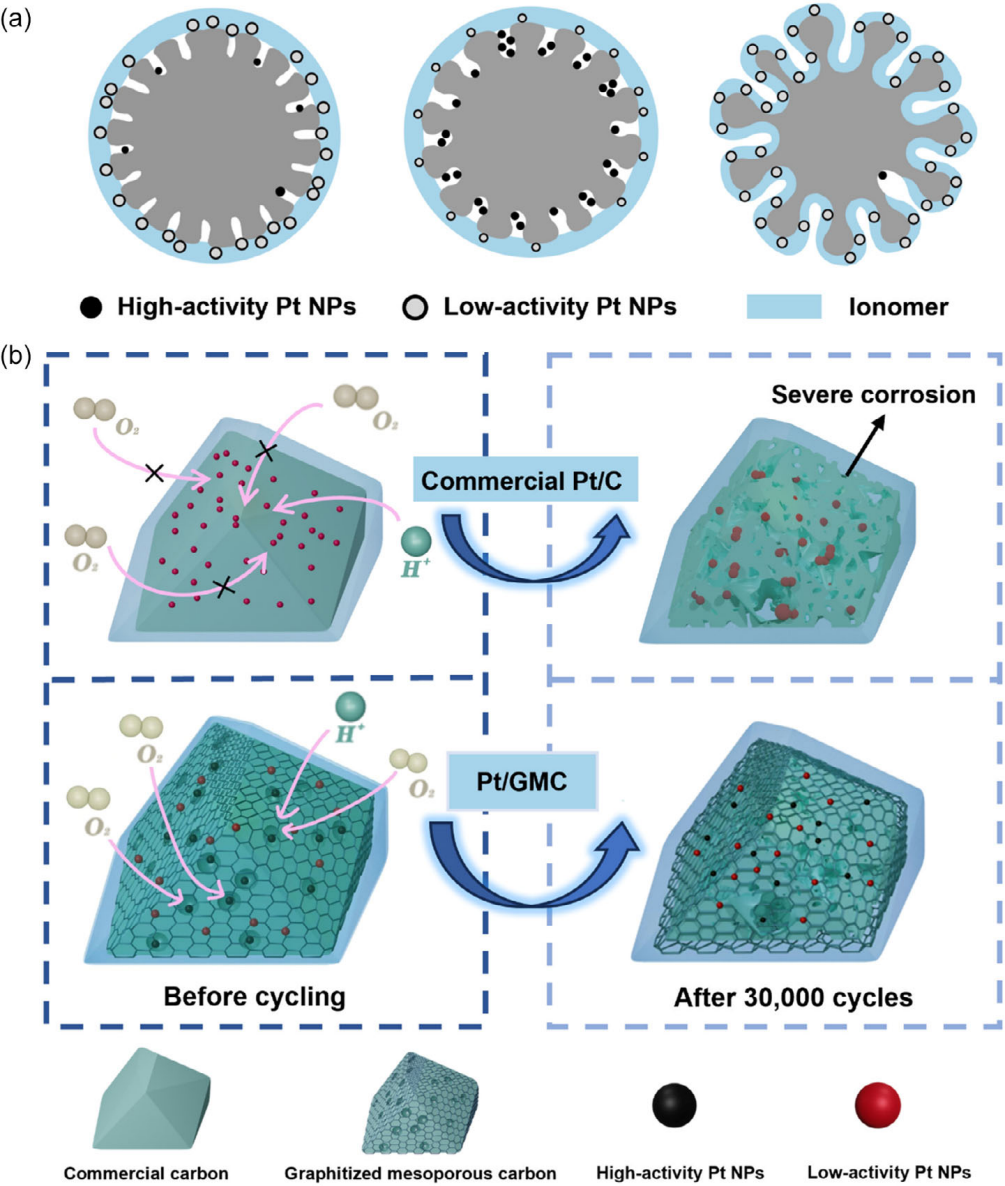
Schematic diagram of a) the impact of carbon support mesopore size on the dispersion of Pt NPs and ionomers. b) Catalyst evolutions schematic diagram before and after attenuation tests.

Based on the above structural characterization and discussion, we developed a schematic diagram for structurally tunable GMC. **Figure** **8**a demonstrates the effect of mesopore size on Pt NPs and ionomers. It should be noted that when the mesopore size of carbon support was too small to allow Pt precursor solution access, most Pt NPs were agglomerated on the outer surface and covered with ionomers. The aqueous solution could enter into the mesopores to maximize support areas with medium‐sized mesopores to avoid Pt NPs agglomerating and ionomer poisoning, the moderate mesopores also facilitate O_2_ transfer and ensure proton conduct via impregnated water inside the mesopores. However, the Pt NPs were aggregated inside the relatively large mesopores with smaller specific surface area, and ionomers could also access such mesopores, resulting in low utilization and activity of Pt NPs. Figure 8b demonstrates the evolution that occurred in carbon support with different structures during the attenuation test. It was observed that the nanostructure of GMC could basically maintain after the attenuation test and the agglomeration degree of Pt NPs was relatively low. In contrast, Pt NPs were highly agglomerated and detached coupled with severe corrosion collapse for amorphous carbon. The ideal carbon support is one with a balance of graphitization and mesopore size that could stably host most Pt NPs inside the mesopores and protect them from sulfonic acid groups adsorption but endow favorable O_2_ and proton accessibility.

## 3 Conclusion

In summary, GMC*x*‐1800 (*x* = 1100, 1300, and 1500) was prepared by in situ formation of tunable nanosized MgO templates under direct high‐temperature pyrolysis, and GMC1300‐1800 exhibited high specific surface area of 1023.2 m^2^ g^−1^ and mesopore size of 7.54 nm. The appropriate mesopores not only protected Pt NPs from sulfonic acid groups poisoning to maximize Pt utilization, but also facilitated O_2_ and proton transport channels to enhance the reactant accessibility. Graphitized carbon support had encouraging corrosion resistance and offered strong interaction between Pt NPs and support, leading to attractive long‐term durability.

Pt/GMC1300‐1800 obtained by platinum nitrate impregnation and 5% H_2_ reduction exhibited uniformly small‐sized Pt NP loading with an average size of 1.5 nm, while the “internal Pt structure ” can be observed in TEM images. Consequently, the initial activity of Pt/GMC1300‐1800 was as high as 106.1 m^2^ g^−1^_Pt_ for ECSA and 0.212 A mg^−1^_Pt_ for MA, 1.6 and 2.1 times those of commercial Pt/C, respectively. After 30 000 cycles of Pt attenuation or carbon attenuation tests, the MA and SA retentions were significantly superior to those of commercial Pt/C, demonstrating the robust durability of Pt/GMC1300‐1800. Meanwhile, the coverage of sulfonic acid groups performed by CO displacement chronoamperometry in MEA fabricated by Pt/GMC1300‐1800 was only 3.8%, much less than other as‐prepared catalysts and commercial Pt/C. The synergistic effect of graphitization and mesoporous structure endows Pt/GMC1300‐1800 with an impressive comprehensive performance, which would provide a promising methodology for future PEMFC technologies with low cost, high efficiency, and robust durability. Additionally, the synthesis of MC support in this article is relatively simple compared to the liquid‐phase synthesis method, but two heating processes were used to obtain the ideal pore size due to the large‐sized MgO NPs and corresponding mesopores, which is a limitation in mass production. Therefore, improving the graphitization process of carbon support may be the focus in our future research work.

## 4 Experimental Section

4.1

4.1.1

##### Preparation of GMC

MgO‐templated MC were synthesized from magnesium citrate according to the literatures.^[^ ^52^ ^]^ Reagent grade of magnesium citrate hydrate (Mg_3_(C_6_H_5_O_7_)_2_·9H_2_O) was heated to 1100, 1300, and 1500 °C under a high‐purity nitrogen atmosphere at a heating rate of 10 °C min^−1^. After holding for 3 h and cooling to room temperature naturally, the products were transformed into carbon‐coated magnesium oxide templates (C@MgO‐*x*, *x* = 1100, 1300, and 1500). MC formed after the removal of the templates by hydrochloric acid etching were denoted as MC‐*x* (*x* = 1100, 1300, and 1500), where the numbers corresponded to heating temperature. GMCs (GMC*x*‐1800, *x* = 1100, 1300, and 1500) were obtained by placing MC‐*x* in a high‐temperature sintering furnace and heating them to 1800 °C under high‐purity argon atmosphere at 5 °C min^−1^ for 1.5 h.

##### Preparation of Carbon‐Supported Pt Catalyst

First, GMC*x*‐1800 was wet milled at 650 r min^−1^ for 36 h using a ball mill to reduce the particle size to less than 500 nm. Then, carbon supports were subjected to liquid‐phase oxidation to change the surface chemical state to generate oxygen surface groups for subsequent Pt loading. All oxidized supports were loaded with 20 wt% Pt through a wet impregnation procedure, in which Pt (NO_3_)_2_ was used as Pt precursor and 5% H_2_/Ar as reducing agent. Pt/GMC*x*‐1800 (*x* = 1100, 1300, and 1500) was performed via the synthesis method based on GMC*x*‐1800. A commercial 20 wt% Pt/C‐JM was used for comparison.

##### Physical Characterization

SEM images were acquired on Zeiss GeminiSEM 500. TEM images were carried out on Talos F200S with energy‐dispersive X‐Ray spectroscopy. The XRD patterns were performed on Bruker D8 with Cu Ka radiation and combined with Scherrer equation to estimate the crystallite sizes. ICP‐MS (Prodigy 7) was used to determine the Pt contents of catalysts. Raman spectroscopy was obtained from LabRAM Odyssey with a laser wavelength of 532 nm. The XPS was confirmed by ESCALAB Xi+. The specific surface area was acquired by nitrogen adsorption/desorption isotherms using the BET method on ASAP 2020, and the adsorption curve was chosen to calculate the pore size distribution.

##### Electrochemical Characterization

A RDE was connected to an electrochemical workstation (Ivium) and electrochemical tests were performed at room temperature using a three‐electrode system. A glassy carbon electrode with an area of 0.19625 cm^2^ was used as the working electrode (WE), reversible hydrogen and Pt wire were used as reference and counter electrodes, respectively, and the electrolyte was HClO_4_ solution (0.1 mol L^−1^). 5 mg of catalyst was added to 500 μL of deionized water, 500 μL of isopropanol and 20 μL of Nafion (5%, DuPont) were added sequentially and dispersed by sonication for more than 30 min to obtain the catalyst ink. After which, a calculated amount (10 μL) of the suspension was then evenly dropped on the clean WE surface and dried at room temperature. Activation of catalyst was acquired on the RDE with a potential interval of 0.05–1.15 V. CV in saturated N_2_ was measured at 100 mV s^−1^ for 120 cycles until the curves essentially overlapped, followed by scanning for one cycle at 50 mV s^−1^, which was treated as the basis for calculating the ECSA. LSV (0.05–1.15 V) for ORR was measured at 1600 rpm under saturated N_2_ with a scanning speed of 10 mV s^−1^ as a background capacitance current at this potential band. Activity test in saturated O_2_ was then carried out at the same rotational speed and scanning rate.

The accelerated attenuation test was conducted using department of energy test standards and divided into two parts: Pt and carbon attenuation tests. The durability of Pt was determined by square waves in a lower potential interval of 0.6–1 V versus reversible hydrogen electrode (RHE). A total of 30 000 cycles were carried out, each cycle taking 4 s. Durability of carbon was obtained by performing 30 000 CV cycles at a higher potential range (1.0–1.5 V vs. RHE). CV (saturated N_2_ at 50 mV s^−1^) and LSV (saturated N_2_, and saturated O_2_ at 10 mV s^−1^) tests were performed at each 10 000 cycles.

Electrochemical properties of the catalysts were acquired by analyzing the changes in ECSA, MA, and SA during the decay process. ECSA was calculated as follows

(1) $$\mathrm{ECSA}=\frac{S_{H}v^{-1}}{0.21\times m_{\text{Pt}}}$$

MA and SA at 0.9 V versus RHE were calculated as follows^[^ ^21^ ^]^

(2) $$\frac{1}{j}=\frac{1}{j_{k}}+\frac{1}{j_{d}}$$

(3) $$\mathrm{MA}=\frac{j_{k}}{m_{\text{Pt}}}$$

(4) $$\mathrm{SA}=\frac{j_{k}}{\mathrm{ECSA}\times m_{\text{Pt}}}$$

where *S*_H_ is the area of the hydrogen evolution peak of Pt NPs, *v* (V s^−1^) is the scan rate of CV, *m*_Pt_ (g) represents Pt loading on the WE, *j* (A m^−2^) is the measured current density at 0.9 V versus RHE, *j*_*k*_ (A m^−2^) is the kinetic current density, and *j*_*d*_ (A m^−2^) is the measured diffusion‐limited current density.

##### MEA Preparation and Single‐Cell Tests

MEAs were prepared using the catalyst‐coated membrane with an active area of 25 cm^2^. Commercial 50 wt% Pt/C was used as the anode catalyst for all experiments with the loading of 0.1 mg_Pt_ cm^−2^. Three as‐prepared catalysts and 20 wt% Pt/C‐JM were used to make an active cathode electrode (loading of 0.15 mg_Pt_ cm^−2^). A certain amount of the catalyst was ultrasonically mixed with 2.21 g of 2‐propanol, 3.32 g of deionized water, and 3.88 g of 5% Nafion solution (DuPont) as the catalyst ink, MEAs were obtained by squeegee coating and hot pressing based on home‐made proton exchange membrane.

Polarization curves of single cells were tested during which pure H_2_ and compressed air were supplied to the anode and cathode sides, a back pressure of 150 kPa and 100% relative humidity was applied on both sides, and the temperature of cells was maintained at 80 °C using a water thermostat. Electrochemical impedance spectroscopy was carried out to evaluate the cell resistance at a current density of 1000 mA cm^−2^ in response to the proton and gas transport resistance.

ECSA was obtained from CV curves. Before conducting experiments, the cathode side was purged with N_2_ until the cell voltage dropped below 0.1 V. CV experiments were carried out at 30 °C, with a scanning range of 0.05–0.95 V and a scanning speed of 50 mV s^−1^. Subsequently, the scanning range was adjusted to 1–1.5 V and ADT was performed for 5000 cycles with the scanning speed increased to 500 mV s^−1^. CV curves were recorded after completing the cycles and the test conditions were adjusted to the initial CV test conditions. Changes in the curves before and after decay can be used to calculate the decay rate of ECSA and determine the durability of catalysts.

The sulfonic acid groups adsorbed on the Pt surface were measured by CO displacement chronoamperometry. A constant temperature and potential (30 °C and 0.54 V) were applied to the cell, which subsequently changed N_2_ on the cathode side to CO. The transient current response (*I–t*) was acquired when a constant potential was applied on the electrode after the addition of CO. Within this process, after the sulfonic acid groups adsorbed on Pt NPs were displaced at a specific potential, the oxidation/reduction current was measured and the displaced charge (*Q*_dis_) was calculated by integrating the current. CO substitution charge (*Q*_strip_) was measured by CV tests after the substitution of CO gas with N_2_. After the CO substitution tests, pure N_2_ was added to the cathode for 30 min before the initial CV measurements. CV measurements were performed in H_2_/N_2_ at 30 °C with a scan rate of 50 mV s^−1^. The following equation was used to calculate the sulfonic acid groups’ coverage on Pt^[^ ^2^ ^]^

(5) $$\text{coverage}=\frac{2Q_{\text{dis}}}{Q_{\text{strip}}}$$

##### Molecular Dynamics Simulations and DFT Calculations

All DFT calculations were realized using the Vienna Ab initio simulation package and the exchange‐correlation potential was depicted by the generalized gradient approximation of Perdew–Burke–Ernzerhof.^[^ ^53^ ^]^ The projector augmented‐wave method was applied to address interactions between ion cores and valence electrons and the planewave cutoff energy was kept constant at 400 eV. Given structural models were relaxed until the Hellmann–Feynman forces were smaller than −0.01 eV Å^−1^, and the energy change less than 10^−5^ eV was attained. To ensure accuracy and reliability, Brillouin zone (grid density) 4 × 4 × 1 was used for GMC material. The formation process of amorphous carbon was based on molecular dynamics simulation of the liquid quenching method, characterized by the simulation of the actual process of amorphous carbon formation in the flame.^[^ ^49^ ^]^ Similar to combustion, during the simulation, amorphous carbon changed from a high‐temperature liquid state to a low‐temperature solid state. Under the regular system canonical ensemble, indicates that a particle with a defined number of particles (N), volume (V), and temperature (T), the total number of simulation steps was set to 10 000, and the running time of each step was 1 fs. Two typical configurations, atomically ordered GMC and disordered amorphous carbon, were constructed to simulate the adsorption behaviors of individual O_2_ molecules and Pt clusters on the carbon surface, respectively. By comparing the magnitude of their adsorption energies on different structural carbons, the most stable structural configuration was identified, thus effectively explaining the effect of the carbon support structure on Pt loading and O_2_ transport.

## Conflict of Interest

The authors declare no conflict of interest.

## Supporting information

Supplementary Material

## Data Availability

The data that support the findings of this study are available from the corresponding author upon reasonable request.
