# Supplementary material for: Structurally Tunable Graphitized Mesoporous Carbon for Enhancing the Accessibility and Durability of Cathode Pt‐Based Catalysts for Proton Exchange Membrane Fuel Cells
Source: Small Sci. 2024 May 19;4(7):2400016. doi: 10.1002/smsc.202400016 (PMC11935204; doi:10.1002/smsc.202400016)
Supplement: Supplementary file 1 — Supplementary Material [file SMSC-4-2400016-s001.pdf]

Structurally tunable graphitized mesoporous carbon for enhancing the accessibility and durability of cathode Pt-based catalysts for PEMFCs

Mingjuan Wu, Zihan Meng[*], Yifei Xiong, Haining Zhang, Aojie Zhang, Hao Zhang, Liyan Zhu, Haibo Tang, Tian Tian, Haolin Tang[*]

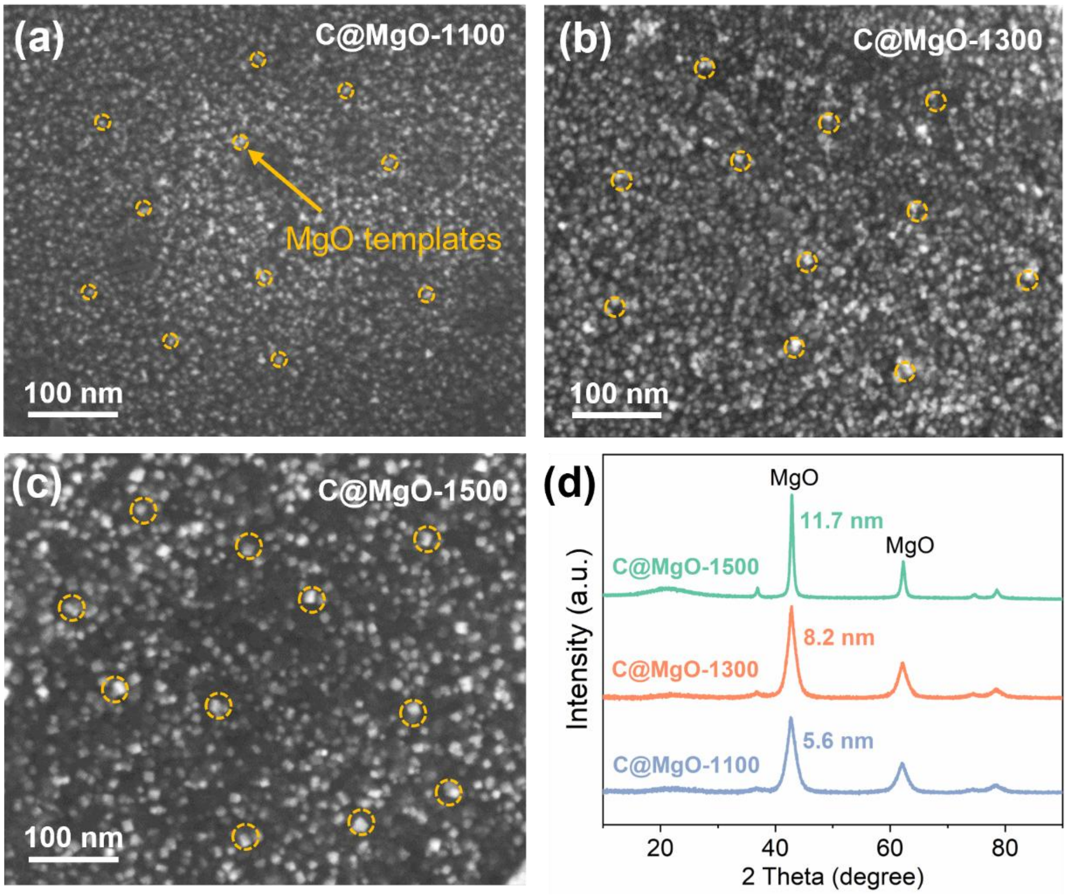

Figure S1. (a-c) SEM images of C@MgO-*x* (*x*=1100, 1300, and 1500), (d) Size of MgO NPs get from XRD patterns of C@MgO-*x*.

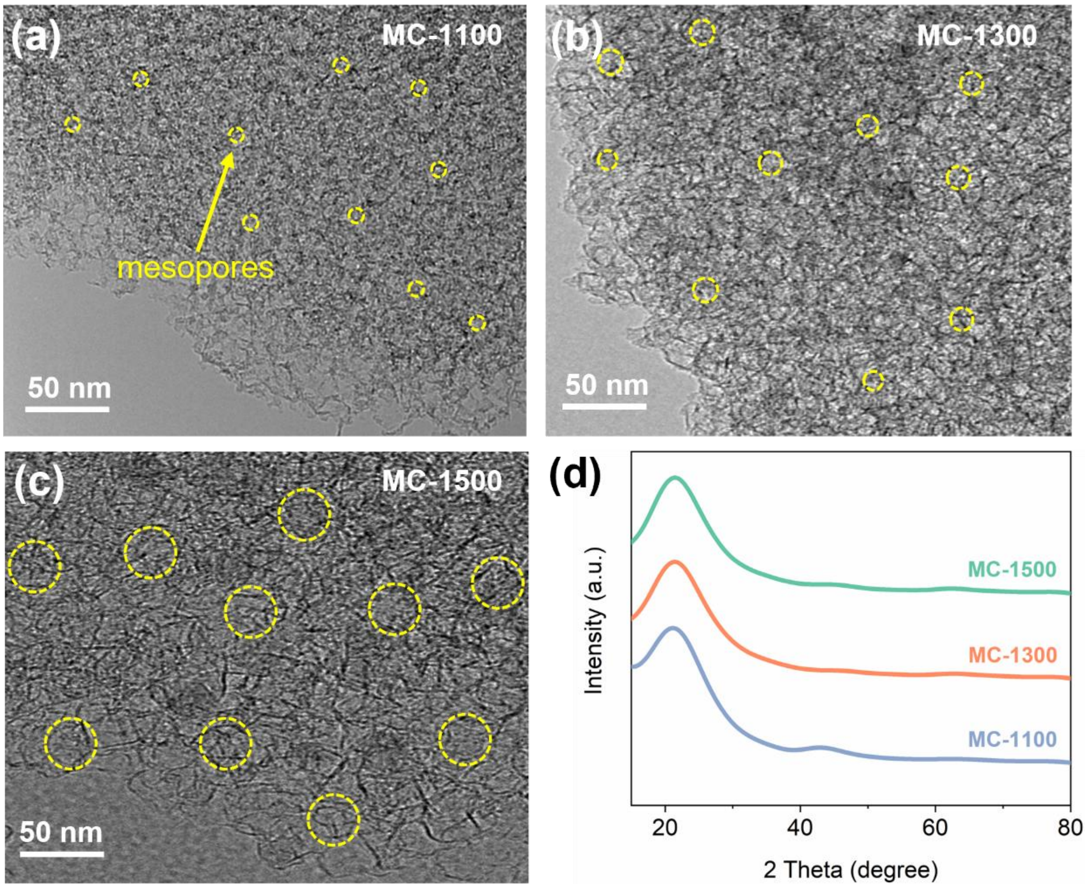

Figure S2. (a-c) TEM images of mesoporous carbon (MC-*x*), (d) XRD patterns of MC-*x* (*x*=1100, 1300, and 1500).

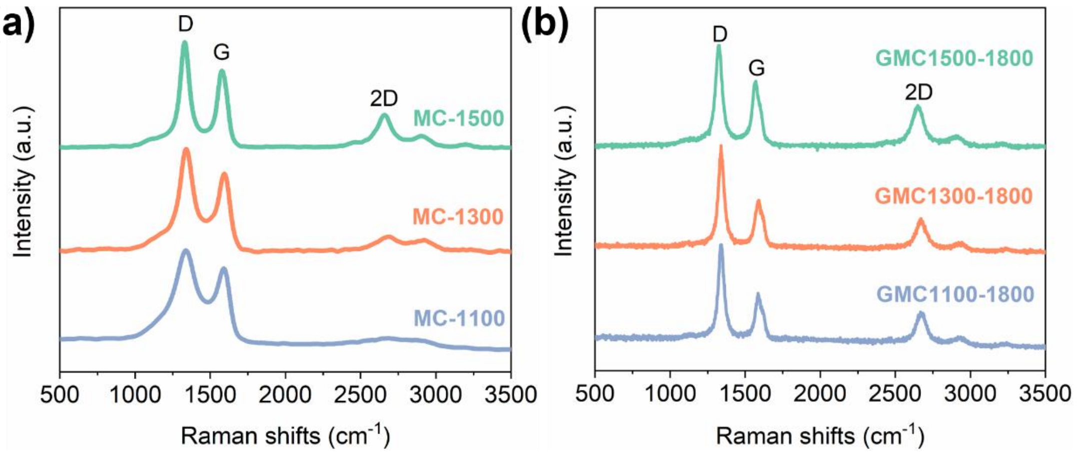

Figure S3. Raman spectra of (a) MC-*x*, and (b) GMC*x*-1800 (*x*=1100, 1300, and 1500).

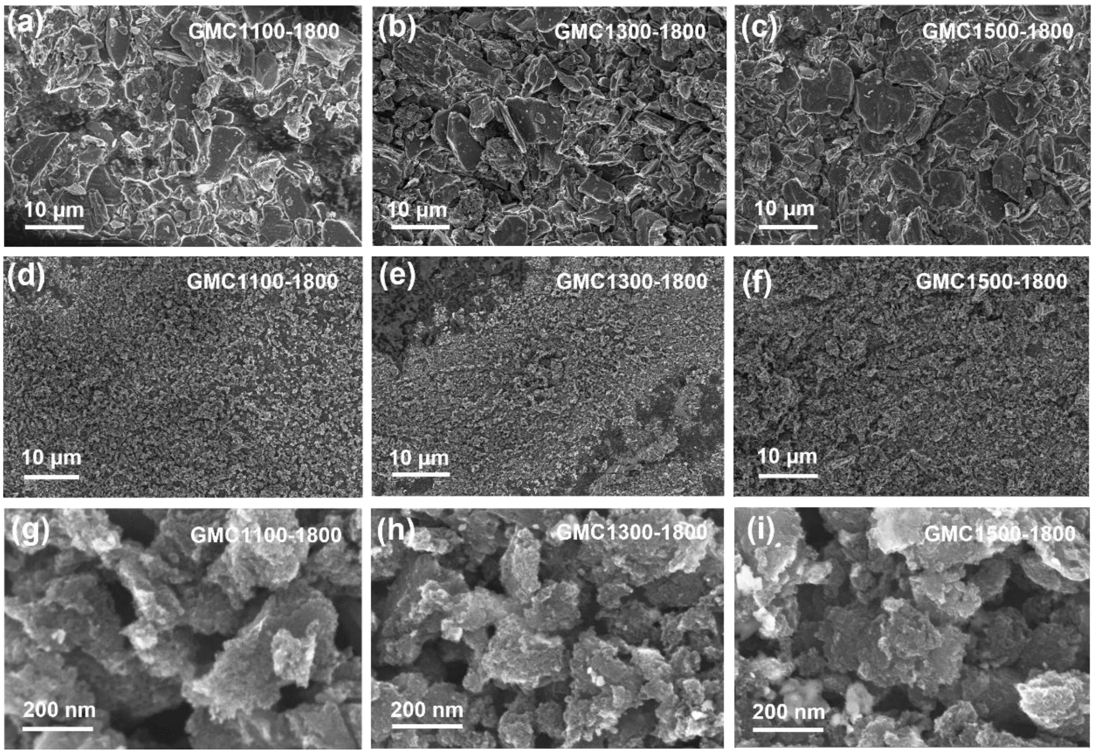

Figure S4. SEM images of (a) GMC1100-1800, (b) GMC1300-1800, and (c) GMC1500-1800 before ball milling. (d-i) Corresponding SEM images of them after ball milling.

Table S1. BET surface area and average pore size of catalysts.

Catalyst	BET surface area (m^2 g^{-1})	Average pore size (nm)
Pt/GMC1100-1800	1134.3	4.61
Pt/GMC1300-1800	1023.2	7.54
Pt/GMC1500-1800	954.8	10.62

Table S2. Platinum loading of the catalysts and size of Pt NPs get from XRD patterns

Catalyst	Pt loading (wt%)	XRD Pt size (nm)
Pt/GMC1100-1800	21.8	2.3
Pt/GMC1300-1800	21.1	1.5
Pt/GMC1500-1800	22.3	2.9

Table S3. The results of ECSA, MA, SA and half-wave potential for catalysts.

Catalysts	ECSA (m^2 g^{-1})	MA at 0.9V (A mg^{-1}_{Pt})	SA at 0.9V (mA cm^{-2}_{Pt})	Half-wave potential (V)
Pt/GMC1100-1800	86.0	0.095	0.110	0.888
Pt/GMC1300-1800	106.1	0.212	0.200	0.918
Pt/GMC1500-1800	76.3	0.050	0.066	0.863
Pt/C-JM	68.5	0.101	0.147	0.899

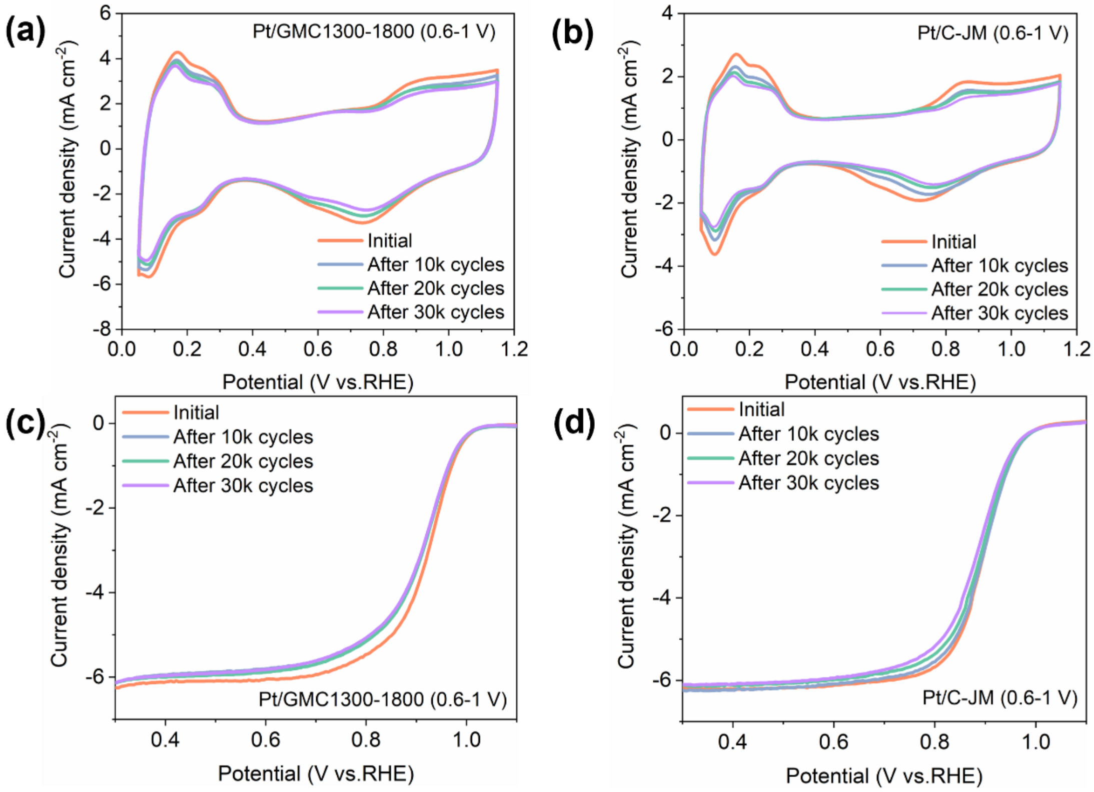

Figure S5. Pt attenuation tests comparison (0.6-1 V): CV curves at 50 mV s^{-1} of (a) Pt/GMC1300-1800 and (b) Pt/C-JM before and after 30,000 cycles (record every 10,000 cycles). LSV curves at 10 mV s^{-1} of (c) Pt/GMC1300-1800 and (d) Pt/C-JM before cycles and after 30,000 cycles (record every 10,000 cycles). Scanning speed during CV cycles: 100 mV s^{-1}.

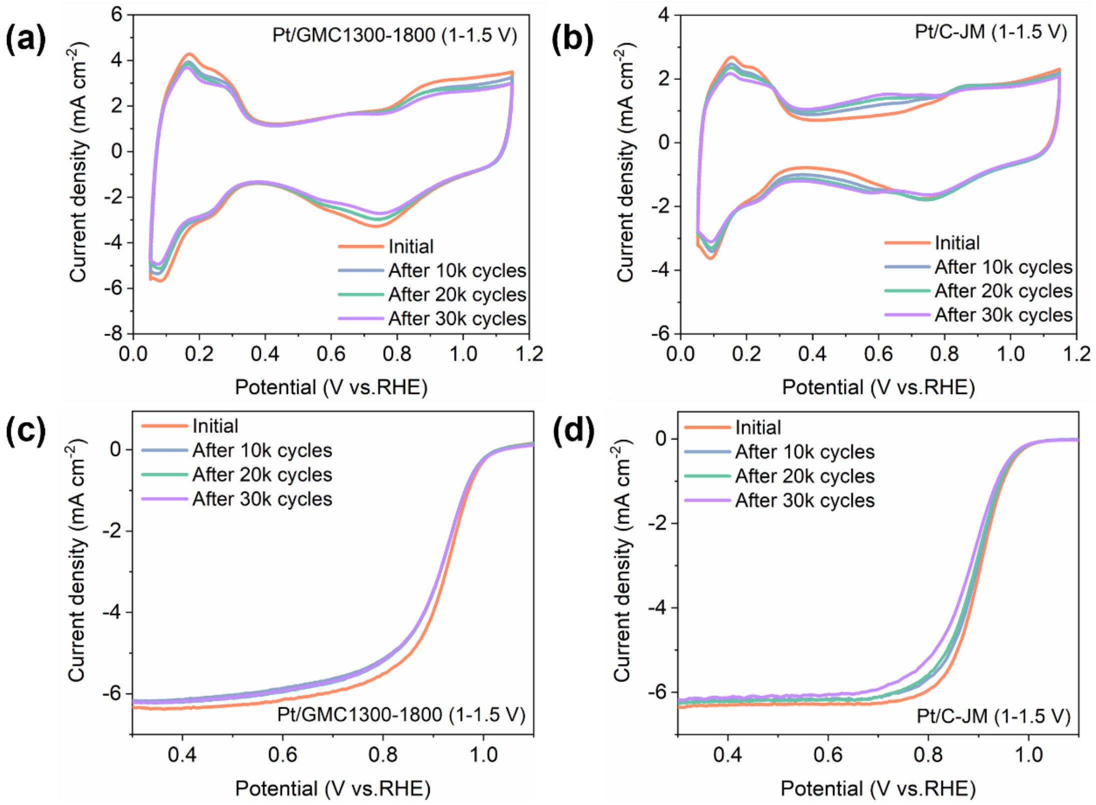

Figure S6. Carbon support attenuation tests comparison (1-1.5 V): CV curves at 50 mV s^{-1} of (a) Pt/GMC1300-1800 and (b) Pt/C-JM before and after 30,000 cycles (record every 10,000 cycles). LSV curves at 10 mV s^{-1} of (c) Pt/GMC1300-1800 and (d) Pt/C-JM before and after 30,000 cycles (record every 10,000 cycles). Scanning speed during CV cycles: 500 mV s^{-1}.

Table S4. Performance comparisons with the state-of-the-art Pt-based ORR catalysts.

Catalysts	ECSA (m^2 g^{-1})	MA at 0.9V (A mg^{-1}_{Pt})	Half-wave potential (V)	Reference
Pt/GMC1300-1800	106.1	0.212	0.918	This work
Pt/C-JM	68.5	0.101	0.899	This work
Pt/CNT	26.9	0.285	0.800	Ruiz-Camacho et al, 2022 [51a]
Hyd-Pt/CS	50.2	0.163	0.850	Saida et al, 2022 [51b]
Pt/HGNC-65	55.7	0.217	0.862	Lee et al, 2023 [51c]
Pt50/CBacid-1200	71.9	0.137	0.923	Zhang et al, 2022 [51d]
C-ZIF-CuPt	80.6	0.240	0.874	Wang et al, 2021 [51e]
Pt/CB	43.0	0.217	0.891	Jiménez-Morales et al, 2021 [51f]
Pt/ Pt-aerogel-ATO	37.5	0.126	0.895	He et al, 2021 [51g]

Table S5. Results of CV and LSV tests before and after ADT for Pt/GMC1300-1800 catalyst.

	Initial	After 30k cycles (0-0.6 V)	After 30k cycles (1-1.5 V)
ECSA (m^2 g^{-1})	106.1	85.6	84.5
MA at 0.9V(A mg^{-1}_{Pt})	0.212	0.147	0.141
SA at 0.9V(mA cm^{-2}_{Pt})	0.200	0.172	0.167
Half-wave potential (V)	0.918	0.913	0.910

Table S6. Results of CV and LSV tests before and after ADT for Pt/C-JM catalyst.

	Initial	After 30k cycles (0-0.6 V)	After 30k cycles (1-1.5 V)
ECSA (m^2 g^{-1})	68.5	46.3	37.3
MA at 0.9V(A mg^{-1}_{Pt})	0.101	0.051	0.041
SA at 0.9V(mA cm^{-2}_{Pt})	0.147	0.110	0.109
Half-wave potential (V)	0.899	0.885	0.883

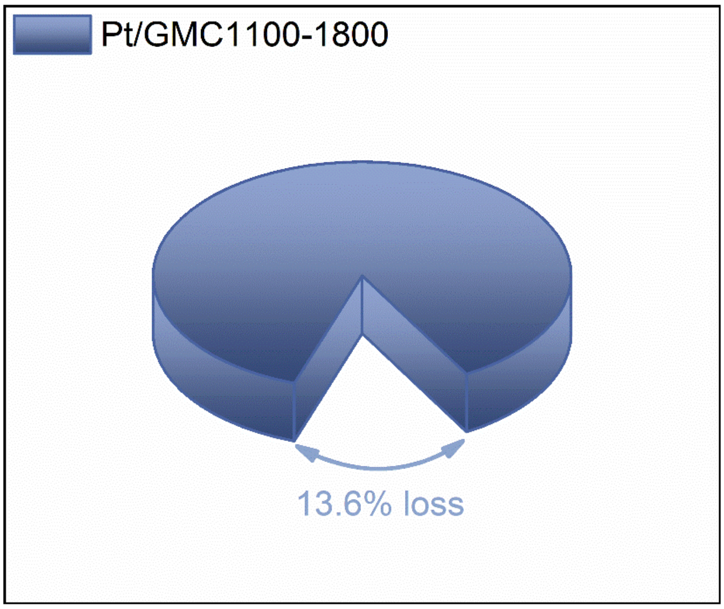

Figure S7. Decay rate of ECSA in MEAs with Pt/GMC1100-1800.

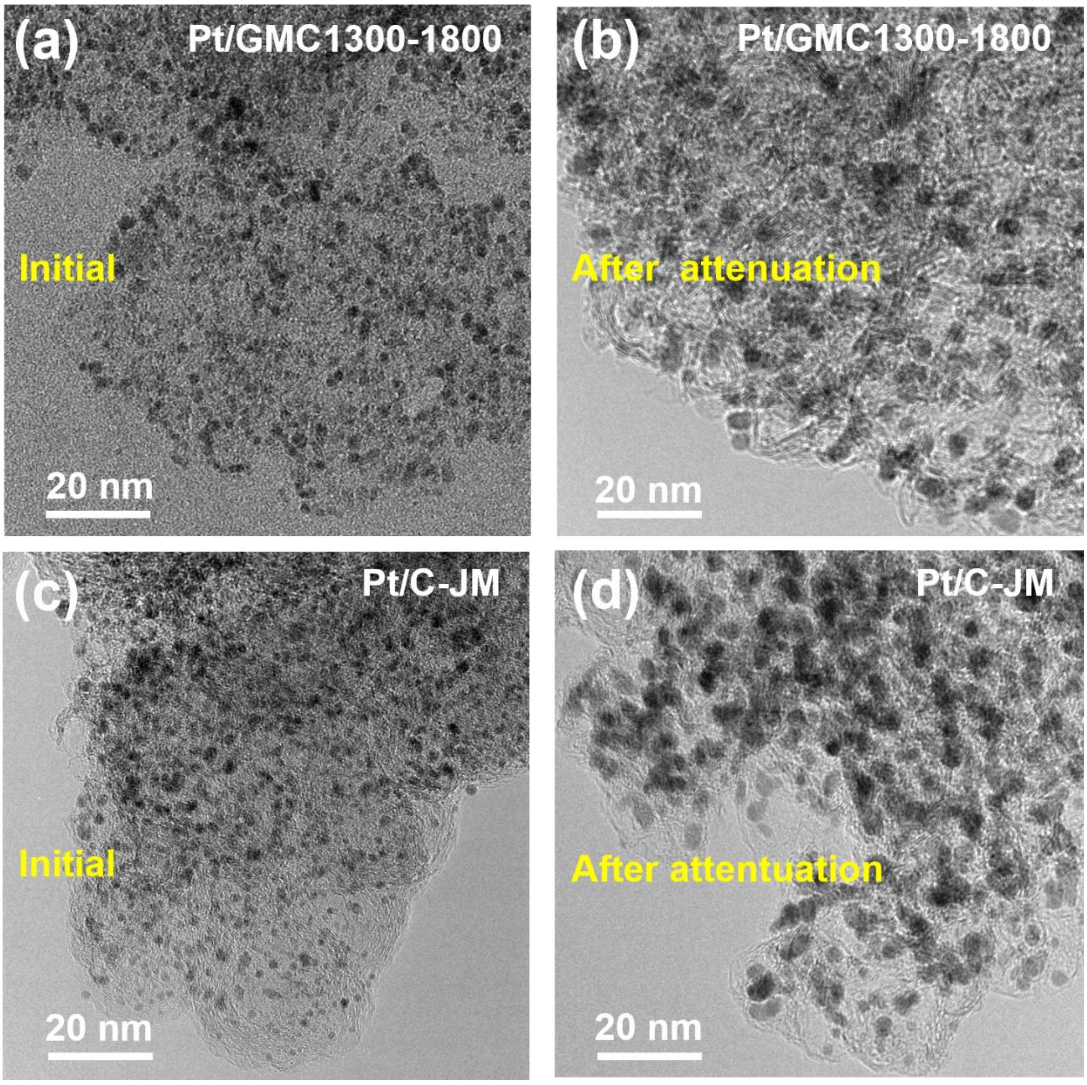

Figure S8. TEM images of (a-b) Pt/GMC1300-1800, and (c-d) Pt/C-JM in MEAs during attenuation over 5000 square wave cycles in the potential range of 1–1.5 V.

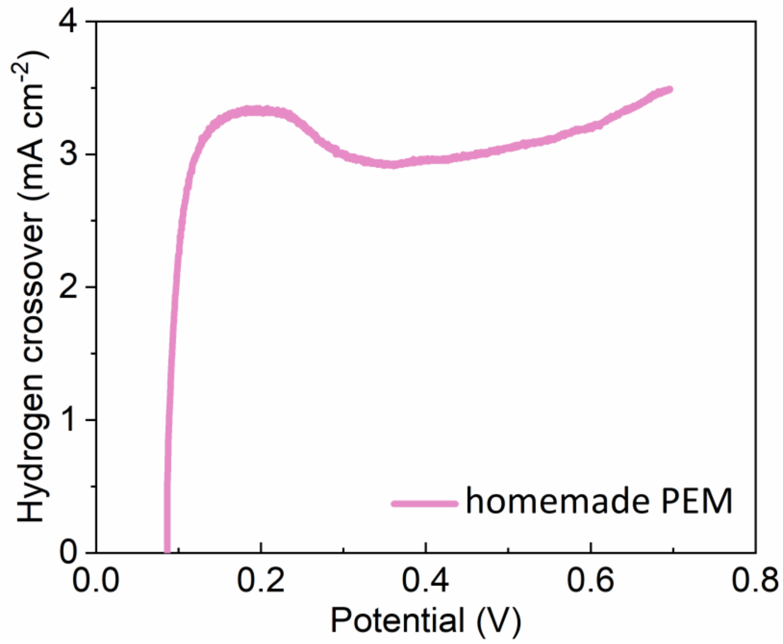

Figure S9. Hydrogen crossover values of the homemade proton exchange membrane.
